# *Cynanchum wilfordii* Etanolic Extract Controls Blood Cholesterol: A Double-blind, Randomized, Placebo-Controlled, Parallel Trial

**DOI:** 10.3390/nu11040836

**Published:** 2019-04-12

**Authors:** Ji Sun Youn, Young Min Ham, Weon-Jong Yoon, Ho-Chun Choi, Ji Eun Lee, Belong Cho, Ji Yeon Kim

**Affiliations:** 1 Department of Food Science and Technology, Seoul National University of Science and Technology, Seoul 01811, Korea; rhdywjd94@naver.com; 2 Biodiversity Research Institute, Jeju Technopark, Seogwipo, Jeju 63608, Korea; hammin81@gmail.com (Y.M.H.); hijel@jejutp.or.kr (W.-J.Y.); 3 Healthcare system Gangnam Center, Seoul National University Hospital, Seoul 06236, Korea; skyho331@gmail.com; 4 Department of Family Medicine, CHA Bundang Medical Center, Seongnam-si 13496, Korea; jieun10@gmail.com; 5 Department of Family Medicine, Seoul National University Hospital, Seoul 03080, Korea

**Keywords:** blood cholesterol, clinical trial, *Cynanchum wilfordii*, LDL-cholesterol, hyperlipidemia

## Abstract

We evaluated the effects of *Cynanchum wilfordii* (CW) ethanolic extract on blood cholesterol levels in adults with high low-density lipoprotein cholesterol (LDL-C) levels. In a double-blind, randomized, placebo-controlled, parallel trial, 84 subjects were recruited. Participants were randomly divided into two groups with a low-dose (300 mg/d) or high-dose (600 mg/d) of CW. Levels of very low-density lipoprotein (*p* = 0.022) and triglycerides (*p* = 0.022) were significantly lower in the low-dose CW group than in the placebo group after 8 weeks. In a subgroup of participants with LDL-C≥ 150 mg/dL (*n* = 33), there was a significant decrease in total cholesterol (low-dose, *p* = 0.012; high-dose, *p* = 0.021), apolipoprotein B (low-dose, *p* = 0.022; high-dose, *p* = 0.016), and cholesteryl ester transfer protein (low-dose, *p* = 0.037; high-dose, *p* = 0.016) after 8 weeks of CW. The correlation between changes in total cholesterol and baseline LDL-C levels was significant in the groups that received both doses of CW (low-dose, *p* = 0.010; high-dose, *p* = 0.015). These results show that the CW ethanolic extract can regulate blood cholesterol in subjects with LDL-C≥ 150 mg/dL.

## 1. Introduction

Cholesterol is an essential component of the body. It influences the fluidity of the cell membrane and is a precursor to steroid hormones. One-third of the total cholesterol (TC) in circulation is derived from food through an exogenous cholesterol transport pathway, and two-thirds is synthesized in the body via an endogenous pathway [1]. Cholesterol synthesized in hepatocytes is transported to the blood in the form of very low-density lipoprotein cholesterol (VLDL-C) by apolipoprotein (apo) E and apoB100. It is transformed into low-density lipoprotein cholesterol (LDL-C) by lipoprotein lipase in blood circulation [2]. LDL-C is a major lipoprotein that carries cholesterol. VLDL-C and LDL-C are both regulated by the enzymes cholesteryl ester transfer protein (CETP) and lecithin cholesterol acyltransferase (LCAT) [3]. CETP catalyzes the hetero-exchange of triglycerides (TG) and cholesteryl esters between plasma lipoproteins [4]; and LCAT, synthesized by the liver, catalyzes the conversion of free cholesterol to cholesteryl esters on high-density lipoprotein cholesterol (HDL-C) and to a lesser degree on LDL-C [5].

Despite this tight regulation, more cholesterol than needed can accumulate in blood vessels causing many diseases. Hyperlipidemia has been associated with high levels of LDL-C and low levels of HDL-C. The number of subjects with hyperlipidemia has been increasing due to diets that are high in saturated or trans-fat. Hyperlipidemia can also result in biliary obstruction, chronic kidney disease, type 2 diabetes mellitus, high blood pressure, and hypothyroidism. Elevated levels of LDL-C can lead to a buildup of plaques within the arteries and increase the risk of cardiovascular disease (CVD), which is a major cause of death due to myocardial infarction and stroke [6]. It was reported that reducing LDL-C levels below 70 mg/dL can effectively lower the risk of CVD in subjects with hypercholesterolemia and diabetic retinopathy. Small dense LDL-C has been reported to be more atherogenic than large buoyant LDL-C [7,8]. Furthermore, a decrease in lipoprotein (a), which consists of LDL-like particles with covalently bound apoB, reduces the risk of CVD [9]. Therefore, many studies have been focusing on the development of novel therapies to control cholesterol in the blood.

*Cynanchum wilfordii* (CW) is found throughout Northeast Asia and its roots have been used in traditional medicines [10]. In Korea, CW is also used for the prevention and treatment of various diseases [11]. Its immune-enhancing effects in macrophages and immunosuppressed mice has been attributed to a crude polysaccharide [12]. The aqueous extracts of CW slowed the progression of testosterone-induced benign prostatic hyperplasia by regulating 5α-reductase and androgen receptor activities in rats [13]. CW has an advantageous effect on osteoporosis by increasing serum osteocalcin concentration [14]. The components of CW include known aromatic compounds such as cynandione A, bungeisides-C, and *p*-hydroxyacetophenone [15]. Cynandione A has been reported to prevent ischemic stroke by regulating the pathway associated with thrombotic or embolic occlusion [16]. Cynandione A also exhibits anti-inflammatory effects by inhibiting VCAM-1 in human endothelial cells or inhibiting MAPK and NF-κB in macrophages [17,18]. Caudatin isolated from CW also exhibits antifungal activity [19]. Caudatin is a target of TNFAIP1/NF-κB signaling, which modulates various carcinogenic processes and affects cell proliferation, migration and apoptosis in uterine cancer cells [20]. Caudatin also inhibits carcinomic human alveolar basal epithelial cell growth and angiogenesis by modulating the GSK3β/β-catenin pathway [21].

Previous studies have reported that CW reduces fat accumulation and liver damage caused by high-fat and high-fructose diets in rats by suppressing COX-2, NF-κB, and p38 MAPK [22]. The ethanolic extract of CW increases blood HDL-C levels and reduces the atherogenic index [23], as well as prevents hypertension and endothelial dysfunction by improving the NO/cGMP signaling pathway in the aortas of rats fed a high-fat and high-cholesterol diet [24]. The ethanolic extracts of CW substantially inhibit the development of atherosclerosis by inhibiting cell adhesion molecules and lesions in apoE^−/−^ mice fed a high-fat and high-cholesterol diet [25]. These studies demonstrate the effect of CW on lipid metabolism on rodents in vivo, but no human clinical studies have been performed. Therefore, we focused on adults with mild hypercholesterolemia to investigate the effects of the ethanolic extract of CW on blood cholesterol levels in humans.

## 2. Materials and Methods

### 2.1. Preparation of CW Extract

The CW used in this study was grown in Jeju-island, Korea. Briefly, the dried powder of CW was extracted with 70% alcohol for 15 h at 60 °C to increase the cynandione A content and extraction yield. Thereafter, the CW powdered extract was concentrated under reduced pressure and lyophilized. The powdered extract was also prepared such that cynandione A, the main component of CW, was at a concentration of 15.0–22.5 mg/g. The test products were prepared to contain 25 or 50% CW ethanol extract powder. The treatment groups were divided into low-dose (CWL, 300 mg/day) and high-dose (CWH, 600 mg/day) groups. Participants were instructed to take two tablets once a day with water. The dose setting was based on a study in which total plasma cholesterol was significantly improved after the administration of 300 mg/kg body weight/day (BW/d) of CW for 4 weeks in an animal model of hyperlipidemia [26]. The dose was calculated by applying the safety factor after converting the human equivalent dose to the average adult weight of 60 kg.

### 2.2. Participants

Adult men and women over 20 years of age were recruited at Seoul National University Hospital for this study. All recruited participants provided written informed consent and had BMIs between 18 and 30 kg/m^2^, LDL-C≥ 130 mg/dL, and TG < 300 mg/dL. The following exclusion criteria were applied: LDL-C ≥ 190 mg/dL; intake of medicines and health functional foods that affected lipid and inflammatory metabolism within 4 weeks of the first visit; weight loss or weight change of 10% or more within 6 months of the first visit; patients suffering from myocardial infarction and stroke; patients with uncontrolled hypertension (systolic blood pressure ≥ 140 mmHg or diastolic blood pressure ≥ 100 mmHg) or diabetes (fasting blood glucose ≥ 180 mg/dL); patients with impaired hepatic function (AST and ALT levels above 2.5 times normal upper limit) or renal function (serum creatinine level ≥ 1.4 mg/dL); patients with chronic inflammatory bowel disease or autoimmune disease; patients with hyperthyroidism, hypothyroidism or malignancy; women under hormonal therapy; excessive smokers (≥20 cigarettes/day); alcoholic or drink more than 140 g/week; performing regular intense exercises (≥10 h/week); participation in another clinical trial within 4 weeks of the first visit; and hypersensitivity to CW.

### 2.3. Study Design

This was a double-blind, randomized, placebo-controlled, and parallel study. After a screening test, the eligible subjects were randomly assigned to the placebo, CWL, or CWH groups, and they consumed the placebo or test food for 8 weeks. Anthropometry and vital signs were measured at weeks 0, 4 and 8. Furthermore, the subjects were assessed for their history of drug and functional food intake. The subjects’ diets were maintained as usual from the run-in period until the end of this study, but some restrictions were in place: foods containing CW and medicines that affect the lipid metabolism in the body, as well as limiting their intake of foods with high cholesterol. The subjects recorded their daily intake of meals, snacks and drinks (excluding water) on a smartphone application developed by BiofoodCRO (Seoul, Korea) for average dietary composition (energy, protein, fat, carbohydrate, and sodium). The protocol was approved by the Institutional Review Board of Seoul National University (IRB No. H-1508-040-694). This study was also registered on the International Clinical Trials Registry Platform of the WHO (ICTRP, No. KCT0001733).

### 2.4. Sample Collection

After the run-in and the intervention periods were completed, fasting blood was collected in Vacutainer blood collection tubes (Becton Dickinson, Franklin Lakes, NJ, USA). The sera centrifuged in serum separation tubes were dispensed in 1.5 mL Eppendorf tubes and stored at −80 °C. Ethylenediaminetetraacetic acid (EDTA) tubes were used for plasma and peripheral blood mononuclear cell collection. First, the plasma was separated from the blood by centrifugation and then stored in 1.5 mL Eppendorf tubes at −80 °C. Peripheral blood mononuclear cells isolated from the buffy coat were washed with cold phosphate buffered saline and stored in RNA stabilization solution (Ambion, Austin, TX, USA) at −80 °C. CETP and LCAT levels in the plasma were analyzed using an enzyme-linked immunosorbent assay (ELISA) kit (Cusabio, Houston, TX, USA).

### 2.5. Statistical Analysis

For the purpose of calculating a sample size (*n =* 28 per group), we referenced a human study that evaluated the efficacy of materials in improving cholesterol in blood [27]. The statistical analysis in this study was performed via a per-protocol method on the subjects who complied with the clinical trial plan and have completed all testing. In the case of non-normal distribution of data, the data were transformed by log transformation or square root transformation and then analyzed by the parametric method. The comparison of subject characteristics at baseline was carried out with ANOVA for continuous variables and Chi-square or Fisher’s exact test for categorical variables. Compliance in the different groups was determined by ANOVA. Dietary intake and physical activity were analyzed using a linear mixed-effect model with group, time, group * time as fixed effect and subject as random effect. The cholesterol-related markers were analyzed by a linear mixed-effect model with group, time, group * time as fixed effect and subject as random effect. The vital signs, hematological test, blood chemical test, and urine test were compared using ANOVA among the groups and paired t-test within each group. In adverse events, Chi-squared or Fisher’s exact test was used to compare the differences in the number of subjects among the groups. The correlation between LDL-C levels at baseline and changes in lipid levels (TC, LDL-C, apoB, CETP and LCAT) were analyzed by Pearson correlation coefficient. The stratified analysis was performed based on the result of correlation analysis. Statistical analysis was performed using SAS (version 9.4; SAS Institute Inc., Cary, NC, USA) and significance was defined as *p* < 0.05.

## 3. Results

### 3.1. Characteristics of Subjects

**Figure 1:**
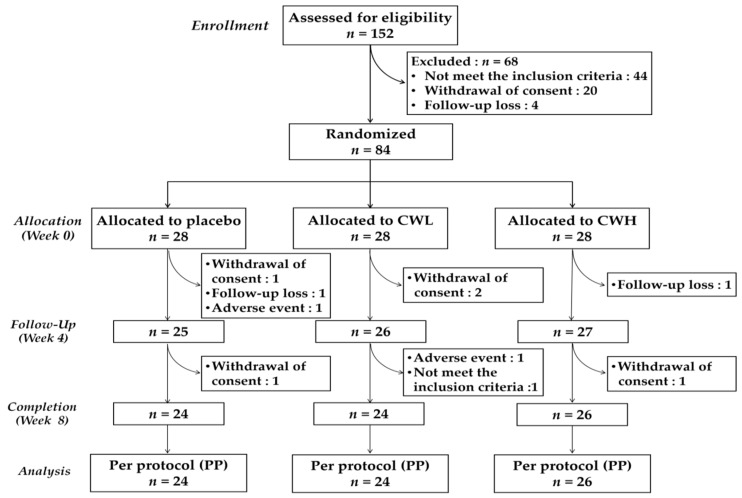
Consolidated Standards of Reporting Trials diagram for flow of participants enrolled in the clinical trial analyzing the cholesterol-controlling effect of *Cynanchum wilfordii*; CWL, low-dose treatment group (300 mg/day); CWH, high-dose treatment group (600 mg/day).

Of the 84 participants, 74 (88.1%) completed the study (Figure 1). After 8 weeks, four subjects dropped out from the placebo (two withdrew consent, one failed to follow-up, and one had an adverse event) and CWL (two withdrew consent, one had an adverse event, and one did not meet the inclusion criteria) groups. In the CWH group, two subjects dropped out, one due to withdrawal of consent and the other failed to follow-up. During the study, 18 adverse events were reported in the placebo (five subjects, five cases), CWL (six subjects, seven cases), and CWH (six subjects, six cases) groups. However, the difference in the number of adverse events was not statistically significant. In addition, no serious adverse event was reported, and symptoms were all mild.

The baseline characteristics of the 74 participants whose data were included in the statistical analyses are provided in Table 1. The study participants had a mean age of 57.9 ± 0.5 years, LDL-C levels of 149.9 ± 2.8 mg/dL, and TC of 232.1 ± 3.1 mg/dL. The proportion of men and women (about 36% and 64%) assigned to each group was similar. There were no significant differences in the baseline characteristics among the groups.

### 3.2. Correlation to Baseline LDL-C

**Figure 2:**
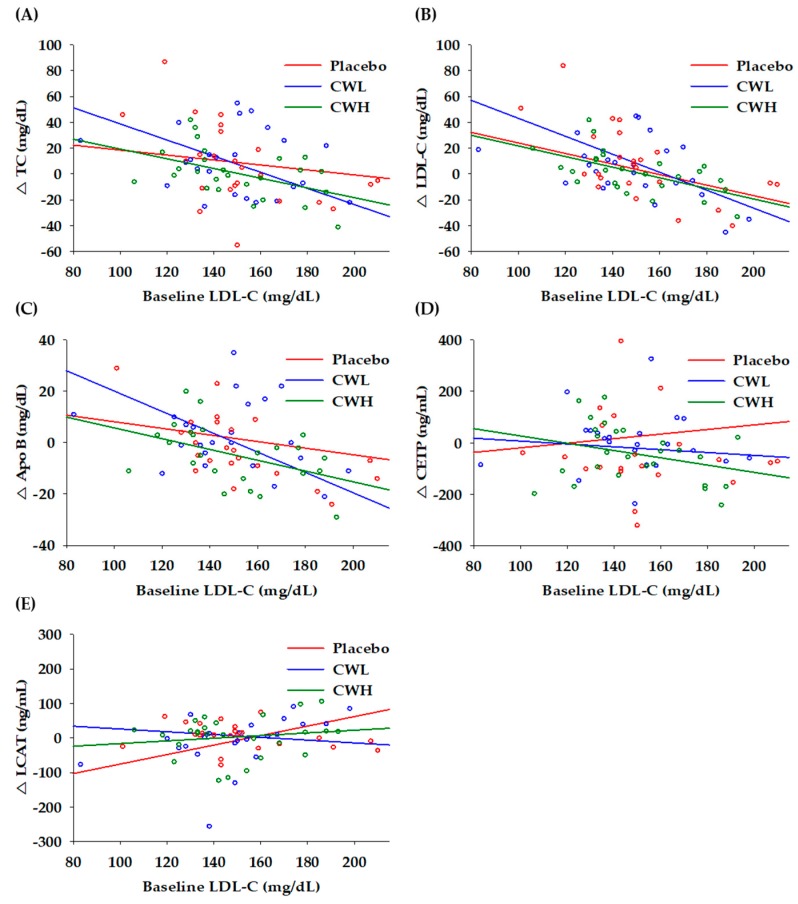
Correlation of cholesterol markers after 8 weeks from baseline LDL-C. (**A**) changes in total cholesterol; (**B**) changes in low density lipoprotein cholesterol; (**C**) changes in apolipoprotein B; (**D**) changes in cholesteryl ester transfer protein; and (**E**) changes in lecithin cholesterol acyltransferase.

The correlation between baseline LDL-C levels and changes in lipid parameters after 8 weeks is shown in Figure 2. The changes in TC showed a negatively significant correlation with baseline LDL-C levels in the CWL (*r* = −0.516, *p* = 0.010) and CWH (*r* = −0.471, *p* = 0.015) groups (Figure 2A), however, there was no significant correlation in the placebo group (*r* = 0.190, *p* = 0.373). The changes in LDL-C were significantly correlated with baseline LDL-C levels in the placebo (*r* = −0.441, *p* = 0.031), CWL (*r* = −0.623, *p* = 0.001), and CWH (*r* = −0.586, *p* = 0.002) groups (Figure 2B). The changes in apoB were also significantly correlated with baseline LDL-C levels in the CWL (*r* = −0.651, *p* = 0.001) and CWH (*r* = −0.442, p = 0.031) groups (Figure 2C). There was no significant correlation between changes in CETP and baseline LDL-C levels (Figure 2D). The changes in LCAT were significantly correlated with baseline LDL-C levels only in the placebo group (r = 0.462, *p* = 0.027) (Figure 2E).

### 3.3. Lipid Profile

**Figure 3:**
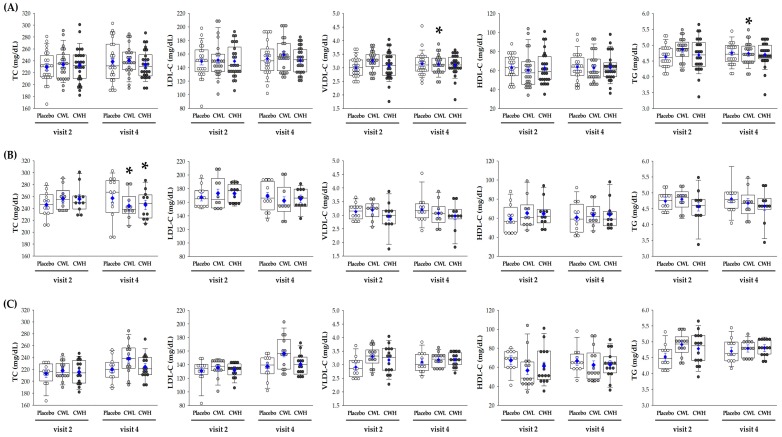
Changes in the lipid profile from baseline to week-8 post treatment. (**A**) Lipid profile analysis of participants (placebo = 24, CWL = 24, CWH = 26); (**B**) high-level LDL-C subgroup (placebo = 12, CWL = 10, CWH = 11) with baseline LDL-C≥ 150 mg/dL; (**C**) low-level LDL-C subgroup (placebo = 12, CWL = 14, CWH = 15) with baseline LDL-C < 150 mg/dL; * *p* < 0.05, Group*time effect between placebo and CW groups.

The lipid profiles of the participants are given in Figure 3A. VLDL-C (*p* = 0.022) and TG (*p* = 0.022) levels were higher in the CWL-treated group than in the placebo group and significantly reduced after 8 weeks. TC and LDL-C levels showed a decreasing trend after 8 weeks in both the high-level and low-level groups; however, there were no significant differences between the groups. Relative to the initial baseline LDL-C recorded, subjects were divided into two subgroups; high-level LDL-C ≥ 150 mg/dL (*n* = 33) and low-level LDL-C < 150 mg/dL (*n =* 41). In the high-level LDL-C subgroup, TC levels in the subjects treated with CWL (*p* = 0.012) or CWH (*p* = 0.021) were significantly reduced after 8 weeks compared to the levels in placebo-treated subjects (Figure 3B). In addition, the group * time effect showed that TC levels (*p* = 0.036) were significantly different among the three groups. LDL-C levels in the CWL group (*p* = 0.032) were significantly reduced after 4 weeks, but there were no statistically significant differences until 8 weeks. However, in the low-level LDL-C subgroups, there were no other statistically significant differences between the groups (Figure 3C).

There were no significant differences in the levels of apo and cholesterol-related enzymes among the groups (Table 2). However, in the high-level LDL-C subgroup, apoB levels in CWL- (*p* = 0.022) and CWH-treated (*p* = 0.016) subjects were significantly reduced after 8 weeks compared to the levels in placebo controls. CETP was also significantly decreased in the CWL (*p* = 0.037) and CWH (*p* = 0.016) groups compared to that in the placebo group. In addition, CETP (*p* = 0.030) levels were significantly different among the three groups based on the group * time effect. In the low-level LDL-C subgroup, apoA1 levels were increased when treated with CWL (*p* = 0.028) compared to those in the placebo group. Furthermore, LCAT levels were significantly increased in the CWL group (*p* = 0.032).

## 4. Discussion

In a previous study, the ability of CW (75, 150 and 300 mg/kg BW/day) to regulate blood cholesterol was evaluated in a hyperlipidemia rat model induced by a high-fat diet. The results showed that TG, VLDL-C and apoB were significantly reduced in CW groups. Furthermore, apoA1 was significantly increased in the CW group. These studies suggested the need for further evaluation of CW in randomized placebo-controlled trials and the possible development of CW supplements for the control of blood cholesterol in vivo.

Similar to a previous animal study, a correlation analysis showed that subjects with higher baseline LDL-C levels benefited greater from CW treatment and significant changes in their lipid profiles was observed. Also, the results of this study showed that TG and VLDL-C levels are significantly reduced by the daily consumption of 300 mg CW for 8 weeks compared to levels in placebo-treated controls. Compared to a high dose, this study found a significant reduction in TG and VLDL-C only at a low dose. Many plant extracts showed hormesis which is the phenomenon where a specific chemical is able to induce biologically opposite effects at different doses [28,29]. In this trial, because dose finding study was not performed, only two doses were compared. In addition, this study focused on TC and the baseline characteristic showed normal TG levels. Therefore, changes in TG and VLDL-C levels should be confirmed in the further trial using subjects with high TG levels. Because of our limited subject size and relatively low LDL-C levels, CW consumption did not show statistical significance in LDL-C reduction after 8 weeks intervention. Based on these results, we hypothesized that CW would have a greater effect on controlling blood cholesterol in subjects with high LDL-C levels. When baseline LDL-C is ≥130 mg/dL, an LDL-lowering drug should be started simultaneously with dietary therapy. For these individuals, clinical management and dietary therapy is recommended when the LDL-C level is ≥160 mg/dL [30]. According to the Adult Treatment Panel III [30], LDL-C level of 150 mg / dL requires an LDL-lowering drug and diet. We performed statistical analysis by dividing the subjects by the subject’s baseline LDL-C average of 150 mg/dL.

Notable is the decrease in apoB, CETP and TC levels due to CW in subjects with baseline LDL-C levels ≥ 150 mg/dL. There was also a significant decrease in LDL-C levels. Similar to our results, consumption of green tea extract for 12 months significantly reduces TC and LDL-C levels in healthy postmenopausal women with baseline TC levels ≥ 200 mg/dL [31,32]. It has also been reported that the supplementation of grape extract with statin-treatment resulted in a decrease in LDL-C for participants with an LDL-C baseline average of 114.2 mg/dL [33]. ApoB synthesis inhibitors have been reported to lower LDL-C levels in hypercholesterolemia subjects with LDL-C baseline average of 243.2 mg/dL [34]. ApoB is a major component of LDL particles and is an apolipoprotein that functions as a lipid accumulator in the blood by dissolving LDL, a fat complex, in the blood [35]. Based on these studies, the significant reduction in apoB level in the treatment groups compared to that in the placebo group was consistent with the effect of CW on blood cholesterol levels. In addition, changes in CETP were concentration-dependent on plasma VLDL levels, and it has been reported that when CETP was inhibited, HDL-C and LDL-C levels changed in a beneficial direction [36,37,38]. These results suggest that CW in the body inhibited the activity of CETP, leading to the inability of VLDL to convert to LDL and consequently to a reduction in the levels of apoB and LDL-C.

In subjects with lower LDL-C levels (<150 mg/dL), LCAT and apoA showed a significant increase only in the CWL group when compared to the placebo group. The ethanolic extract of CW increases blood HDL-C levels and reduces the atherogenic index although there was no significant difference in total cholesterol in the high-cholesterol diet SD rats and apoE^−/−^ mice [23]. In this study, CW was standardized with cynandione A which was reported to have anti-inflammatory effects by inhibiting VCAM-1 in human endothelial cells or inhibiting MAPK and NF-κB in macrophages [17,18]. Besides cynandione A, CW has been identified to have various phytochemicals such as dihydroxyacetophenone, acetovanillone, conduritol F, geniposide, bungeiside A and β-sitosterol [15,39]. Among these compounds, β-sitosterol has been reported to upregulate paraoxonase-1 (PON1) [40]. PON1 binding to HDL through apoA attracted significant interest for its potential for the most antioxidant properties of HDL [41]. A few dietary flavonoids including anthocyanin and grape polyphenols increased PON1 and LCAT activities and resulted in an increase in HDL levels [42,43]. This result showed the possibility for the effect of CW at a low dose on increasing HDL-C levels, however, subjects participated in this study had relatively healthy HDL-C levels and the significant result was only at a low dose [44]. In addition, this subgroup, because of relatively healthy cholesterol levels, there were no significant changes in TC or LDL-C levels. Our correlation analysis showed significant positive correlation between baseline LDL-C levels and changes in LCAT only in the placebo group. None the less, the inconsistency in the result of LCAT and apoA1 in the subgroup of lower LDL-C level is the major limitation of the current study. Therefore, a further mechanism study for the elevation of HDL-C levels and the effect of CW on LCAT activity should be elucidated.

The correlation analysis also showed that changes in blood cholesterol due to CW intake were more effectively controlled in participants with LDL-C levels of 150 mg/dL or more than in those with slightly higher LDL levels than normal. TC levels decreased only in the subjects with higher LDL-C levels. Although LCAT and apoA1 increased significantly in the CWL group during subgroup analysis, HDL-C levels were not changed by CW supplementation. This suggests that CW might be effective in subjects with higher LDL levels. CVD, a major cause of death, has an important relationship with blood cholesterol, especially LDL-C levels. Previous studies have demonstrated a 20% reduction in CVD risk by lowering LDL-C levels to 38.67 mg/dL. Bjorck et al. concluded that the largest reduction in death was explained by substantial reductions in total cholesterol levels, resulting in a 39% of the mortality reduction [45,46]. CETP, an important regulator of plasma lipoprotein, appeared to be directly related to atherosclerosis in several studies [47,48]. Considering the above studies, our study suggests the possibility for effect of CW intake on maintaining normal blood cholesterol levels in adults with high LDL levels. However, in order to be developed as an alternative for statin therapy, other clinical trials targeted hyperlipidemia patients and more detailed mechanism studies should be performed.

Unfortunately, although sample size was calculated based on a previous human study that evaluated the efficacy of materials in improving cholesterol in blood, statistical significance was not reached in the major endpoints such as TC or LDL-C levels by CW supplementation. A significant difference was found in the results only in stratification analysis, this might be due to the relatively small number of subjects in each subgroup as well as the relatively healthy blood cholesterol levels. Accordingly, clinical studies with higher blood cholesterol levels or LDL-C levels that are large enough to obtain statistical reliability are needed in the next study. In addition, because of the limitations associated with in vitro and animal studies, active compounds have not been identified. Nevertheless, this study might be worth noting because it is the first clinical study that evaluates the effect of CW on hyperlipidemia. This study also identified the safety of CWE supplementation as well as its efficacy. During the study, several adverse events were reported such as discomfort in urinary function, allergic reaction, vomiting, problems with skin, and gastrointestinal discomfort. All of these events were mild reactions and there was no statistical significance among treated groups. Our findings in this study show the possibility that CW may be developed as food supplements for helping maintain blood cholesterol levels in people with high levels of LDL cholesterol.

## Figures and Tables

**Table 1:** Baseline characteristics of the 74 study participants

Variables	Placebo ^1^ (*n* = 24)	CWL ^1^ (*n* = 24)	CWH ^1^ (*n* = 26)	*p*-Value ^2^
Gender (male/female)	8/16	9/15	10/16	0.924
Age (y)	58.1 ± 2.3	57.4 ± 1.8	58.3 ± 1.8	0.940
Body weight (kg)	60.9 ± 2.5	64.0 ± 2.3	62.8 ± 1.8	0.630
BMI (kg/m^2^)	23.6 ± 0.6	24.8 ± 0.6	23.9 ± 0.5	0.257
Dietary intake				
Energy (kcal/d)	1343.5 ± 81.7	1483.7 ± 72.2	1374.9 ± 72.3	0.398
Carbohydrate (g/d)	201.8 ± 12.5	217.3 ± 14.0	209.9 ± 13.4	0.718
Protein (g/d)	52.5 ± 3.4	60.5 ± 3.6	61.2 ± 11.2	0.632
Fat (g/d)	35.0 ± 3.2	39.7 ± 3.3	32.5 ± 2.5	0.242
Sodium (mg/d)	2628.7 ± 233.8	2832.4 ± 241.5	2692.0 ± 217.4	0.818
SBP (mmHg)	119.8 ± 2.4	124.3 ± 2.1	121.4 ± 1.8	0.338
DBP (mmHg)	71.5 ± 1.8	74.3 ± 1.7	74.1 ± 1.7	0.467
Blood				
TC (mg/dL)	229.5 ± 5.4	233.9 ± 5.3	232.7 ± 5.7	0.844
LDL-C (mg/dL)	149.0 ± 5.0	151.2 ± 5.3	149.4 ± 4.6	0.948
HDL-C (mg/dL)	63.2 ± 2.7	60.2 ± 3.8	62.7 ± 3.3	0.796
VLDL-C (mg/dL)	21.8 ± 1.6	27.5 ± 1.9	24.7 ± 2.4	0.135
TG (mg/dL)	108.9 ± 8.1	137.3 ± 9.4	123.3 ± 12.2	0.135

^1^ Mean ± SE; ^2^ ANOVA for continuous variables and Chi-square or Fisher’s exact test for categorical variables were used to compare the difference among the groups.

**Table 2:** Changes in lipid parameters through a linear mixed-effect model used to analyze group, time and group*time effects after 8 weeks.

	Total					Baseline LDL-C≥ 150 mg/dL					Baseline LDL-C< 150 mg/dL				
Variables	CWL ^1^ (*n* = 24)	*p* ^2^	CWH ^1^ (*n* = 26)	*p* ^3^	*p* ^4^	CWL ^1^ (*n* = 10)	*p* ^2^	CWH ^1^ (*n* = 11)	*p* ^3^	*p* ^4^	CWL ^1^ (*n* = 14)	*p* ^2^	CWH ^1^ (*n* = 15)	*p* ^3^	*p* ^4^
**LP (a)** (mg/dL)															
Week 4	3.15 ± 3.75	0.403	−0.25 ± 3.65	0.945		6.07 ± 5.38	0.264	0.84 ± 5.14	0.871		0.74 ± 5.33	0.890	−1.33 ± 5.25	0.800	
Week 8	4.13 ± 3.78	0.276	2.50 ± 3.68	0.498	0.759	−0.76 ± 5.45	0.889	−0.06 ± 5.22	0.991	0.690	8.10 ± 5.33	0.133	4.83 ± 5.25	0.360	0.545
**ApoA1** (mg/dL)															
Week 4	1.75 ± 4.88	0.720	0.83 ± 4.85	0.864		−10.73 ± 6.57	0.108	−7.47 ± 6.52	0.257		12.39 ± 6.82	0.073	8.73 ± 6.79	0.203	
Week 8	2.92 ± 4.88	0.551	2.21 ± 4.85	0.649	0.982	−12.67 ± 6.57	0.059	−6.85 ± 6.40	0.289	0.351	15.31 ± 6.82	0.028	10.39 ± 6.87	0.135	0.222
**ApoB** (mg/dL)															
Week 4	−5.62 ± 3.90	0.151	−2.58 ± 3.88	0.506		−10.82 ± 5.53	0.055	−6.00 ± 5.49	0.278		−1.51 ± 5.22	0.773	0.87 ± 5.19	0.868	
Week 8	−2.04 ± 3.90	0.601	−6.64 ± 3.88	0.089	0.202	−13.05 ± 5.53	0.022	−13.39 ± 5.39	0.016	0.069	6.08 ± 5.22	0.247	−0.87 ± 5.25	0.869	0.382
**ApoE** (mg/dL)															
Week 4	−0.25 ± 0.24	0.307	−0.29 ± 0.24	0.224		−0.31 ± 0.34	0.368	−0.18 ± 0.34	0.599		−0.18 ± 0.34	0.592	−0.35 ± 0.34	0.304	
Week 8	−0.36 ± 0.24	0.137	−0.31 ± 0.24	0.194	0.547	−0.52 ± 0.34	0.134	−0.62 ± 0.33	0.068	0.367	−0.18 ± 0.34	0.606	−0.02 ± 0.34	0.952	0.767
**CETP** (ng/mL)															
Week 8	−46.15 ± 50.08	0.360	−67.55 ± 48.61	0.169	0.375	−134.20 ± 61.51	0.037	−149.68 ± 58.22	0.016	0.030	25.00 ± 75.71	0.743	4.29 ± 74.59	0.954	0.935
**LCAT** (ng/mL)															
Week 8	13.25 ± 17.36	0.448	10.76 ± 16.85	0.525	0.719	−28.21 ± 21.37	0.197	−16.15 ± 20.23	0.431	0.419	55.15 ± 24.81	0.032	42.66 ± 24.44	0.089	0.085

^1^ Estimate ± SE; ^2^ Group * time effect between placebo and CWL group; ^3^ Group * time effect between placebo and CWH group; ^4^ Group, time and group * time effects among the placebo, CWL and CWH groups; LP(a), lipoprotein(a); ApoA1, apolipoprotein A1; ApoB, apolipoprotein B; ApoE, apolipoprotein E; CETP, cholesteryl ester transfer protein; LCAT, lecithin cholesterol acyltransferase.
